# Honey and metformin ameliorated diabetes-induced damages in testes of rat; correlation with hormonal changes

**Published:** 2013-12

**Authors:** Ozra Nasrolahi, Fereshteh Khaneshi, Fatemeh Rahmani, Mazdak Razi

**Affiliations:** 1 *Department of Biology, Faculty of Sciences, Urmia University, Urmia, Iran.*; 2 *Department of Comparative Histology and Embryology, Faculty of Veterinary Medicine, Urmia University, Urmia, Iran.*

**Keywords:** *Diabetes*, *Honey*, *Metformin*, *Spermatogenesis*, *Testis*

## Abstract

**Background:** The global prevalence of diabetes mellitus is on rise. Diabetes-induced oxidative stress has been known to affect liver, pancreas, kidney and reproductive organs pathologically. Honey is a natural product of bee with antioxidant properties.

**Objective: **Current study aimed to analyze the protective effects of Metformin (MF) alone and MF+ natural honey co-administration on diabetes-induced histological derangements in testis of rats.

**Materials and Methods:** Thirty six, mature male Wistar rats were randomly divided into six groups including; control, honey-dosed non-diabetic, diabetes-induced (65 mg/kg, single dose), honey-administrated diabetic (1.0 g/kg/day), Metformin-received diabetic (100 mg/kg/day), Metformin and honey-co-treated diabetic which were followed 40 days. The animals were anesthetized by diethyl ether and the blood samples were collected. The serum levels of testosterone, Insulin, LH and FSH analyzed using antibody enzyme immunoassay method. The testicular tissues were dissected out and underwent to histological analyses.

**Results:** The biochemical analyses revealed that the diabetes resulted in significantly reduced testosterone (p<0.01), LH and FSH (P<0.01, 0.001) levels in serum. Light microscopic analyses showed remarkable (p<0.01) reduction in seminiferous tubules diameter (STD), spermiogenesis index (SPI) and thickness of the epithelium in the diabetic group versus control and co-treated groups. Simultaneous administration of the honey with MF could fairly up-regulate testosterone, LH and FSH levels. The animals in metformin and honey-treated group exhibited with improved tubules atrophy, elevated spermiogenesis index and germinal epithelium thickness.

**Conclusion:** Our data indicated that co-administration of Metformin and honey could inhibit the diabetes-induced damages in testicular tissue. Moreover, the simultaneous administration of metformin and honey up-regulated the diabetes-reduced insulin, LH, FSH and testosterone levels.

This article extracted from M.Sc. thesis. (Ozra Nasrolahi)

## Introduction

Diabetes mellitus is known as a common public health problem and it has been identified as one of the five global causes of death (1). Previous reports showed that intensified oxidative stress following increased blood glucose plays an important role in creating of diabetes-induced complications (2). Severe reduction in serum concentration of testosterone beside negative impact on reproductive system as; remarkable reduction in accessory sex glands weight, reduced epididymal sperm content and increased thickness of basement membrane are reported for diabetic patients (3, 4). According to previous experiences the diabetes-induced derangements are not controlled by administrating unique compound, indicating several impacts of diabetes. 

Furthermore, the available drugs have undesirable side effects such as hypoglycemic and diabetes complications associated with oxidative stresses (5). Therefore, using antioxidant compound in order to prevent and/or delay oxidative stress-dependent degeneration seems to be more logic (6). The beneficial effects of antioxidants in preventing or ameliorating testis damage in rodents have been shown in some studies (7). Honey is a natural product of bees and it contains of different compounds such as carbohydrates, conventional minerals, proteins, vitamins, organic acids, enzymes and antioxidants such as catalase, peroxidase, alkaloids, polyphenols and flavonoids (8). Beside all above mentioned, the honey exerts many medically beneficial effects including; hypoglycemic, antioxidant, hepatoprotective, reproductive, antihypertensive effects (5, 6, 9, 10). 

Other studies clarified that honey reduced the level of lipid peroxidation in the testis of rats exposed to cigarette smoke and it may ameliorate oxidative stress in the gastrointestinal tract (GIT), liver, pancreas, kidney, reproductive organs and plasma/serum (6, 11). Also the synergistic antioxidant effect of honey with antidiabetic drugs in the pancreas, kidney and serum of diabetic rats has been reported (6). Metformin is a normal hypoglycemic agent and a drug regulating blood sugar. Its main task is to increase insulin sensitivity in liver and facilitating the transport of glucose in hyperglycemia and insulin resistance (12). Although metformin has been known for a choice used-compound for poly cystic ovarian syndrome, on the other hand its potential harmful impact on gonads growth has been reported in male fetuses (13, 14). 

Herein we minded that the administrating a compound with antioxidant properties associated with one substance which is able to induce the hypoglycemia can control the diabetes-inducing damages. Therefore in current study, the effect of natural honey (as an antioxidant) co-administration with MF (as a hypoglycemic drug) against diabetes-induced damages was investigated. 

## Materials and methods

**Animals**

This study is an original experimental research. To follow-up present study, 36 mature Wistar male rats (5 weeks old), weighing 200±20g were purchased from Pasteur Institute of Tehran and acclimatized in an environmentally control room for one week (12h light/12h dark cycle at temperature of 25±2^o^C). The rats were fed with standard food and tap water *ad libitum. *All experiments were conducted in accordance with the Institutional Guidelines for the Care and Use of Animals for Experimental Purposes of Urmia University.

**Induction of diabetes**

Animals were off fed for 12 hours. Then diabetes was induced by intraperitoneal administration of Streptozotocin (STZ, Sigma, U.S.A.). Three days after STZ injection (65 mg/kg b.w), development of diabetes was confirmed by measuring glucose level in fasting blood samples taken from tail vein using Accu-Chek glucometer (Roche, Germany). Rats with blood glucose concentrations of 220 mg/dl or higher were considered diabetic and included in the study. Blood glucose levels of the control rats remained normal (<100 mg/dl). 

**Compounds preparation**

Honey was obtained from forests of Ilam province and its naturality confirmed by the Agricultural Research center of Ilam. Physicochemical properties of honey were measured according to harmonized methods of the International Honey Commission (Bogdanov, 1999) and National Iranian Standard No. 92 (honey-Specifications and test methods). Honey contained 0.986% of total reducing sugar including [fructose (35.56%), glucose (30.04%); fructose/glucose ratio (0.99)], sucrose (1.14%), water (17.5%), Hydroxymethyl furfural (HMF) (3/40mg/kg), Radical scavenging activity (57/25% inhibition) and Antioxidant activity of 328/32 μM Fe (II). The honey (1.0 g/kg b.w) was freshly dissolved with distilled water just before each administration. MF (HEXAL, Germany) 100 mg/kg b.w. was dissolved in distilled water before administration (5).

**Animal treatment**

Thirty six mature male Wistar rats were divided into six groups (N=6 in each group) as control and test groups. The test group subdivided into 5 groups as;

1. Diabetes-induced (D); a single dose of STZ was administrated (65 mg/kg., ip).
2. Honey-received diabetic group (HD); received 1 gr/kg/day honey orally by gavage.
3. Metformin-administrated diabetic group (MD); received 100 mg/kg metformin by oral gavage.
4. Honey and metformin co-treated diabetic group (HMD).
5. Non-diabetic-honey-administrated group (HC).

The control group (C) received0.5ml of saline normal. All compounds and vehicles were administrated for 4 weeks. The animals were off-feeded for 16 hours and then the tissue and blood samples were collected. 

**Serum sampling**

Blood samples were collected directly from heart in centrifuge tubes without anticoagulants and allowed to clot. The clotted blood was then centrifuged at 3000 g for 10 min. Serum was separated and then quickly stored at -80^o^C for biochemical analyses

**Evaluating serum levels of FSH, LH, Testosterone and insulin**

The serum levels of luteinizing hormone (LH) and follicular stimulating hormone (FSH) were assayed using the method of double antibody enzyme immunoassay as described in the kits purchased from Biosource, Belgium (FERTIGENIX-FSH-EASIA code 40 170 12, FERIGENIX-LH-EASIA, code 40 131 00, and FERTIGENIX-FSH-EASIA code 40 084 00).Testosterone was assessed by using competitive chemiluminescent immunoassay kit (DRG Co, Germany; and Pishtaz Teb, Iran). The insulin was estimated by ELISA method. 

**Histological analysis**

Analysis of the diameter of seminiferous tubules was done with calibrated lens using Sudaman method. In this method, the average of the small and big diameter of each tubule was calculated using the formula magnification (15).

$\sqrt[{2}]{\mathrm{small diameter}\times \mathrm{large diameter}\times \mathrm{magnification}}$

The thickness of the epithelium was calculated starting from basic membrane until spermatids based on micrometer (16). To evaluate spermatogenesis index (SPI), 200 seminiferous tubules in one section from each animal were scored for sperm present in each tubule. A tubule was scored for spermatogenesis if it contained sperm (17).

**Statistical analysis**

Statistical analysis was carried out using SPSS 16 software (SPSS/PC-16, SPSS Inc, USA). The data were expressed as Mean±SEM which was used for ANOVA analysis and Tukey post Hoc test. The p<0.05 was considered statistically significant.

## Results

**Biochemical changes**

The biochemical analyses manifested that, the animals in HMD, HC and MD groups, showed significantly (p<0.01) higher serum level of testosterone in comparison to those in diabetes-induced group (Table I). The serum level of LH was significantly (p<0.01) decreased in the diabetic control rats (D) compared to non-diabetic rats. Treatment with MF or honey significantly increased the LH level in diabetic rats. The animals in HMD group showed significantly (p<0.001) higher serum level of LH in comparison to those in diabetes-induced group. No significant effect of honey in non-diabetic rats was observed (Table I).

Administration of HMD or HD significantly (P<0.01) increased the levels of FSH in diabetic rats compared to diabetic control rats and diabetic rats treated with MF alone (Table I). The diabetic control rats had significantly (p<0.001) reduced insulin level compared to non-diabetic rats. Treatment of diabetic rats with honey and Metformin as well as their combinations produced a significant increase in insulin levels compared to diabetic controls (Table I).

**Histological observations**

Histological analyses showed that the animals in group D exhibited remarkable (p<0.01) reduction in seminiferous tubules diameter, while co-treatment with honey and metformin significantly elevated diabetes-decreased tubular diameter. The data for morphometric analyses are presented in (Table II). The percentage of tubules with positive SPI significantly (p<0.01) reduced in group D versus to group C. In contrast, the treated (HD and HMD) groups manifested with increased percentage of tubules with positive SPI (Table II). More light microscopic analyses revealed that the germinal epithelium thickness significantly decreased in non-treated diabetic group. Meanwhile the treated animals were exhibited improved germinal epithelium thickness. Comparing the interstitial edema between different test groups showed that, the honey and metformin-simultaneous administration inhibited the diabetes-induced edema (Table II). 

**Figure 1:**
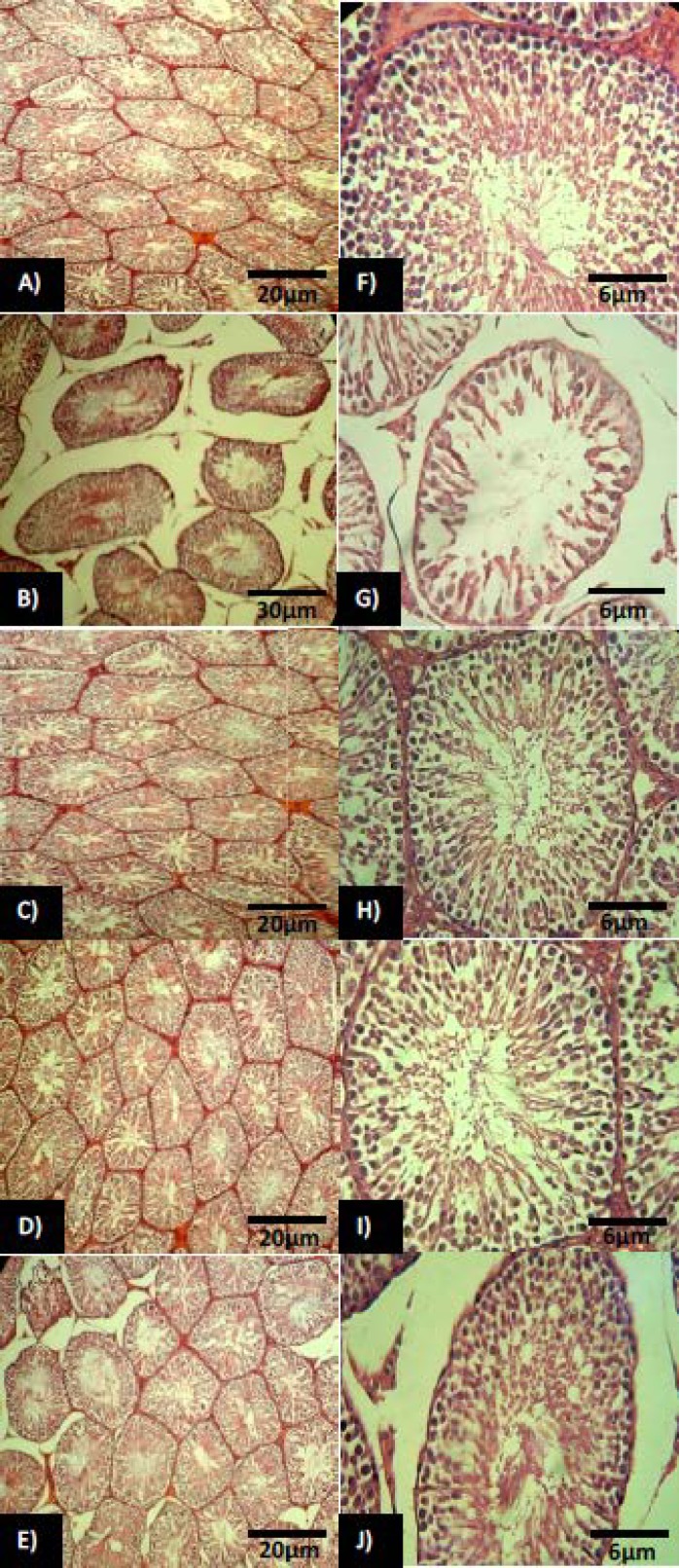
Cross section from testis; (**A**) control group: note normal testicular tissue with normal seminiferous tubules in higher magnification (**F**). (**B**) Non-treated diabetic testis: the seminiferous tubules are degenerated and remarkable edema is manifested in interstitial tissue. Note negative TDI and SPI in higher magnification (**G**). (**C**) Metformin and honey co-treated group, showing normal spermatogenesis (**H**) with decreased edema. (**D**) Honey alone-treated group: The tubules are appeared approximately normal accomplished with significant decrease in edema. Note the normal spermatogenesis in higher magnification (**I**). (**E**) Metformin alone-treated group: The tubules are presented with higher thickness of germinal epithelium (**J**) while the mild edema remained in interstitial tissue. H&E staining technique, (**A, B, C, D, E**: 100× and **F, G, H, I, J**: 400×).

Texture studies showed that in C and HC groups, the seminiferous tubules and the germinal epithelium are completely healthy in terms of appearance and all levels of spermatogenic cells (Figure 1-A, 1-F). However, light microscopic analyses showed that the germinal epithelium of the diabetic rats underwent to a severe degeneration which was manifested with remarkable reduction in germ cells height. Moreover observations demonstrated that the germinal cells dissociation increased in diabetic rats which it was accomplished with dislocations in several tubules. Increased interstitial tissue expansion with remarkable edema between tubules observed in diabetes-induced rats. 

Animals in MD and HMD groups were manifested with remarkable reduction in seminiferous tubules degeneration, increased germinal epithelium height and significant improvement in cellular junction (Figure1-C, 1-H, 1-E, 1-J). In diabetic group treated by honey (HD), all seminiferous tubules were normal with increased germinal epithelium height and improvement in cellular junction and all levels of spermatogenic cells (Figure 1-D, 1-I). Diabetic group that was treated by metformin (MD) also showed increased germinal epithelium height and seminiferous tubules compared to diabetic control group.

**Table I:** Effects of natural honey, Metformin or their co-administration on serum levels of testosterone, LH, FSH and Insulin in different groups (N=6 rats per group).

**Group**	**Testosterone (ng/ml)**	**LH (IU/L)**	**FSH (IU/L)**	**Insulin (pmol/L)**
C	3.60 ± 0.04^a^	0.23 ± 0.002^a^	0.13 ± 0.004^a^	2.18 ± 0.014^a^
HC	3.57 ± 0.04^a^	0.22 ± 0.002^b^	0.12 ± 0.002^b^	2.18 ± 0.014^a^
D	2.55 ± 0.06^b^	0.14 ± 0.004^c^	0.05 ± 0.004^c^	0.95 ± 0.017^b^
MD	3.16 ± 0.004c	0.18 ± 0.004d	0.08 ± 0.002d	1.74 ± 0.018c
HD	3.21 ± 0.004d	0.19 ± 0.004e	0.12 ± 0.002e	2.12 ± 0.018d
HMD	3.25 ± 0.004d	0.19 ± 0.004e	0.13 ± 0.002f	2.175 ± 0.012d

D: Diabetes-induced (65 mg/kg)

MD: Metformin (100 mg/kg)-received

HD: Honey-administrated (1 gr/kg/day)

HMD: honey and metformin-co-treated.

All data are presented as Mean±SE

a, b, c, d, e, f present significant differences (p<0.01, p<0.001) between marked data in same column.

**Table II:** Effects of natural honey, Metformin or co-administration on seminiferous tubules diameter (STD), spermiogenesis index (SPI) and thickness of the epithelium in different groups (N=6 rats per group).

**group**	**STD (µm)**	**SPI (%)**	**thickness of the epithelium (µm)**
C	225.0 ± 6.4^a^	95.5 ± 0.6^a^	45.0 ± 0.4^a^
HC	227.50 ± 4.7^a^	95.5 ± 0.2^a^	45.2 ± 0.4^a^
D	159.2 ± 4.1^b^	57.0 ± 0.9^b^	30.0 ± 0.4^b^
MD	190.5 ± 0.4^c^	84.5 ± 0.6^c^	38.7 ± 0.2^c^
HD	192.2 ± 0.8^c^	94.0 ± 1.2^d^	37.7 ± 0.4^c^
HMD	203.7 ± 1.0^d^	97.0 ± 0.8^d^	39.5 ± 0.2^d^

D: Diabetes-induced (65mg/kg),

MD: Metformin (100mg/kg)-received

HD: Honey-administrated (1gr/kg/day)

HMD: honey and metformin-co-treated.

All data are presented as Mean±SE

a, b, c, d present significant differences (p<0.01) between marked data in same column.

## Discussion

The deterioration of pancreatic β-cell function is caused by low expression of antioxidant enzymes such as superoxide dismutase, catalase, and glutathione peroxidase in the pancreas and increased generation of reactive oxygen species (ROS). Antioxidants have been identified as protection to the pancreas against oxidative stress in diabetes mellitus. Previously it has been shown that honey could fairly decrease the oxidative stress-induced damages in testicular tissue following smoking (11). Considering this finding, it may be concluded that the phenolic compounds such as flavonoids as a powerful antioxidants are able to prevent and/or inhibit diabetes-induced derangements (6). 

On the other hand, honey contains different minerals such as zinc, selenium, copper, calcium, potassium, chromium, manganese. Some of these minerals have play vital roles in the maintenance of normal glucose tolerance, insulin secretion from the pancreas and some else involved in glucose and insulin metabolism (5). Thus here in this study the honey was administrated in order to protect the testicular tissue against diabetes-exerted damages in testicular tissue by two mechanisms of honey’s antioxidant feature and its effect on blood level of glucose. Present study showed that the blood level of insulin decreased in diabetic rats, which was in good accordance with those findings with Erejowa *et al*, whereas the diabetic animals which were received honey (HD) manifested with improved levels of insulin. This improvement could be related to honey’s protective effect on β-cells of pancreas (10).

It has been illustrated that, creating of any experimental diabetes in rats is associated with disorder in reproductive system physiologic function in both genders (18). Indeed, the diabetes impacts on the testicular tissue due to insufficient production of insulin which in turn results in reducing the sertoli and leydig cells endocrine function (19). Moreover the diabetes-decreased serum level of FSH can severely affect the testicular endocrine function and spermatogenesis as well. In the light of this hypothesis, previous reports showed that decreased level of insulin in diabetic rats resulted into remarkable reduction in FSH level (19). 

Our biochemical analyses exhibited that the serum levels of FSH and LH decreased in diabetic rats. Meanwhile the rats in HD and HMD groups showed improved levels of gonadotrophic hormones. Minding that the insulin is essential for sustain receptors on leydig cells and for leydig cells division, we can suggest that honey alone (HD) and in combination with MF (HMD) could protect spermatogenesis by up-regulating the insulin level in the blood (20). This hypothesis approved by our biochemical analyses, as the animals in treated groups showed significantly increased levels of insulin. 

Studies showed that oxidative stress reduces enzymatic and non-enzymatic level in leydig cells and causes reduction of testosterone. Therefore, the diabetes-induced oxidative stress in testicular tissue inhibits androgenesis by leydig cells (21). In present research the serum level of testosterone reduced in diabetic group which was in accordance with the findings of Stefanovic *et al* (22). We found that the animals in treated groups showed significant increased in testosterone level. As any derangement in testosterone synthesis can impact the spermatogenesis negatively, the honey, albeit with some differences, could up-regulate the testosterone level and subsequently protected spermatogenesis cell lineage (23).

It is well noted that there is a negative correlation between increased ROS level and normal spermatogenesis (24). It has been shown that the diabetes results in remarkable reduction in spermatogenesis and decreases the seminiferous tubules diameter by tubular atrophy (25). Histological observations revealed that the animals in HD and HMD groups manifested with positive spermiogenesis index. 

Considering the fact that the normal spermatogenesis largely depends on lowered oxidative stress and increased endocrine activity by leydig and sertoli cells, we can conclude that honey can decrease tubular atrophy partly by down-regulating oxidative stress and as well by improving testosterone biosynthesis. Thus, the animals in treated groups exhibited remarkably higher germinal epithelium height and as well the seminiferous tubules diameter in comparison to non-treated animals. 

## Conclusion

Our data suggest that, the honey could inhibit the diabetes-induced damages in testicular tissue. Moreover, honey and metformin co-administration showed better results versus other forms of application. Thus it could be suggested that simultaneous administration of honey with metformin could be considered as appropriate form of application, as the testes of honey-received groups were manifested with improved histological features. Moreover, honey and metformin could improve testicular endocrine activities partly by regulating gonadotropins levels.

## References

[1] Erejuwa OO, Sulaiman SA, Wahab MS, Sirajudeen KNS, Salleh MS, Gurtu S (2011). Effect of Glibenclamide alone versus Glibenclamide and Honey on Oxidative Stress in Pancreas of Streptozotocin Induced Diabetic Rats. Int J Appl Res Nat Prod.

[2] Rösen P, Nawroth PP, King G, Möller W, Tritschler HJ, Packer L (2001). The role of oxidative stress in the onset and progression of diabetes and its complications: asummary of a Congress Series sponsored by UNESCO-MCBN, the American Diabetes Association and the German Diabetes Society. Diabetes Metab Res Rev.

[3] Balasubramanian K, Sivashanmugam P, Thameemdheen S, Govindarajulu P ( 1991). Effect of diabetes mellitus on epididymal enzymes of adult rats. Indian J Exp Biol.

[4] Rohrbach DH, Martin GR ( 1982). Structure of basement membrane in normal and diabetic tissue. Ann NY Acad Sci.

[5] Erejuwa OO, Sulaiman SA, AbWahab MS, Sirajudeen KNS, Salleh MS, Gurtu S (2011). Glibenclamide or Metformin Combined with Honey Improves Glycemic Control in Streptozotocin-Induced Diabetic Rats. Int J Biol Sci.

[6] Erejuwa OO, Sulaiman SA, Mohd Sab (2012). Honey: A Novel Antioxidant. Molecules.

[7] Agarwal A, Prabakaran SA, Said TM ( 2005). Prevention of oxidative stress injury to sperm. J Androl.

[8] Gheldof N, Wang XH, Engeseth NJ ( 2002). Identification and Quantification of Antioxidant Components of Honeys from Various Floral Sources. J Agric Food Chem.

[9] Erejuwa OO, Sulaiman SA, Wahab MS, Salam SK, Salleh MS, Gurtu S (2012). Hepatoprotective effect of tualang honey supplementation in streptozotocin-induced diabetic rats. Int J Appl Res Nat Prod.

[10] Erejuwa OO, Sulaiman SA, Wahab MS, Sirajudeen KNS, Salleh MS, Gurtu S (2011). antioxidant protection of Malaysian tualang honey in pancreas of normal and streptozotocin-induced diabetic rats. Ann Endocrinol (Paris).

[11] Mohamed M, Sulaiman SA, Jaafar H, Sirajudeen KN (2011). Antioxidant protective effect of honey in cigarette smoke-induced testicular damage in rats. Int J Mol Sci.

[12] Wu MS, Johnston P, Sheu WH, Hollenbeck CB, Jeng CY, Goldfine ID ( 1990). Effect of metformin on carbohydrate and lipoprotein metabolism in NIDDM patients. Diabetes Care.

[13] Moll E, Veen F, Wely M ( 2007). The role of metformin in polycystic ovary syndrome: a systematic review. Hum Reprod.

[14] Tartarin P, Moison D, Froment P (2012). Metformin exposure affects human and mouse fetal testicular cells. Hum Reprod.

[15] Soudmany S, Yuvaraj S, Malini T, Balasubramanian K (2005). Experimental diabetes has adverse effects on the differentiation of ventral prostate during sexual maturation of rats. Anat Rec A Discov Mol Cell Evol Biol.

[16] Khayatnouri MH, Safavi SE, Safarmashaei S, Mikailpourardabili B, Babazadeh D ( 2011). Effect of Saffron on Histomorphometric Changes of Testicular Tissue in Rat. Am J Anim Vet Sci.

[17] Shetty G, Wilson G, Huhtaniemi I, Shuttlesworth GA, Reissmann T, Meistrich ML ( 2000). Gonadotropin-releasing hormone analogs stimulate and testosterone inhibits the recovery of spermatogenesis in irradiated rat. Endocrinology.

[18] Orth JM, Murray FT, Bardin CW ( 1979). Ultrastructura Changes in Leydig Cells of Streptozotocin-induced Diabetic Rats. Anat Rec.

[19] Kühn-Velten N, Waldenburger D, Staib W ( 1982). Evaluation of steroid biosynthetic lesions inIsolated Leydig Cells from the Testes of Streptozotocin-Diabetic Rats. Diabetologia.

[20] Kiani fard D, Hasanzadeh SH, Sadrkhanluo RA, Farshid M (2011). An investigation of ultrastructural changes in cells of seminiferous tubules of testes and alterations in gonadotropic-gonadal hormones of adult male experimentally induced diabetic rats. Urmia Med J.

[21] Bairyk L, Kumar G, Yeshwanth R ( 2009). effect of acyclovir on the sperm parameters of albino mice. Indian J Physiol Pharmacol.

[22] Stefanovic A, Stevuljevic JK, Spasic S, Stanojevic NB, Bujisic N (2008). The influence of obesity on the oxidative stress status and the concentration of leptin in type 2 diabetes mellitus patients. Diabetes Res Clin Pract.

[23] Yang J, Zhang Y, Wang Y, Cui S ( 2007). Toxiceffects of zearalenone and alpha-zearalenol on the regulation of steroidogenesis and testosterone production in mouse Leydig cells. Toxicol In Vitro.

[24] Said TM, Aziz N, Sharma RK, Lewis-Jones I, Anthony J, Thomas Jr (2005). Novel association between sperm deformity index and oxidative stress-induced DNA damagein infertile male patients. Asian J Androl.

[25] Guneli E, Tugyan K, Ozturk H, Gmustekin M (2008). Effect of melatonin on testicular damage in Streptozotocin-induced diabetic rats. Eur Surg Res.

